# Long-term stability predictions of therapeutic monoclonal antibodies in solution using Arrhenius-based kinetics

**DOI:** 10.1038/s41598-021-99875-9

**Published:** 2021-10-15

**Authors:** Drago Kuzman, Marko Bunc, Miha Ravnik, Fritz Reiter, Lan Žagar, Matjaž Bončina

**Affiliations:** 1 Biologics Drug Product, Technical Research and Development, Global Drug Development, Novartis, Lek D.D., Kolodvorska 27, 1234 Mengeš, Slovenia; 2 Faculty of Mathematics and Physics, University of Ljubljana, Ljubljana, Slovenia; 3 Josef Stefan Institute, Ljubljana, Slovenia; 4 Regulatory Affairs CMC, Global Drug Development, Novartis, Sandoz GmbH, Kundl, Austria; 5 Revelo d.o.o., Ljubljana, Slovenia

**Keywords:** Biological physics, Biophysical chemistry, Chemical physics, Computational models, Drug development

## Abstract

Long-term stability of monoclonal antibodies to be used as biologics is a key aspect in their development. Therefore, its possible early prediction from accelerated stability studies is of major interest, despite currently being regarded as not sufficiently robust. In this work, using a combination of accelerated stability studies (up to 6 months) and first order degradation kinetic model, we are able to predict the long-term stability (up to 3 years) of multiple monoclonal antibody formulations. More specifically, we can robustly predict the long-term stability behaviour of a protein at the intended storage condition (5 °C), based on up to six months of data obtained for multiple quality attributes from different temperatures, usually from intended (5 °C), accelerated (25 °C) and stress conditions (40 °C). We have performed stability studies and evaluated the stability data of several mAbs including IgG1, IgG2, and fusion proteins, and validated our model by overlaying the 95% prediction interval and experimental stability data from up to 36 months. We demonstrated improved robustness, speed and accuracy of kinetic long-term stability prediction as compared to classical linear extrapolation used today, which justifies long-term stability prediction and shelf-life extrapolation for some biologics such as monoclonal antibodies. This work aims to contribute towards further development and refinement of the regulatory landscape that could steer toward allowing extrapolation for biologics during the developmental phase, clinical phase, and also in marketing authorisation applications, as already established today for small molecules.

## Introduction

Generation, evaluation, and interpretation of stability data of a pharmaceutical product are key aspects of drug development^1,2^. During early technical development, stability data inform several important decisions, such as formulation selection, the selection of primary packaging materials or defining the manufacturing process. In addition, stability data are the basis for setting the shelf-life of a product, i.e. the period in which the quality of a product has to remain within pre-set and well-justified shelf-life specifications. Obtaining real-time stability data is very time-consuming and is a bottleneck of early technical development. Speeding up the process with accelerated conditions (e.g. higher temperature) has been therefore attempted many times but with limited success in accurate prediction^3–8^. Therefore, achieving robust and reliable long-term prediction of stability (usually several years) from accelerated stability (usually a few months) is of major scientific and applied interest. Establishing robust prediction approaches could allow for use of accelerated and stress conditions to determine the shelf-life (safety) of a product beyond the period covered by actually measured and tested stability data.

Biologics like monoclonal antibodies, bispecific antibodies and fusion proteins are susceptible to a variety of chemically- and temperature-induced structural changes^2^. For example, chemical changes of amino acid residues, such as deamidation of asparagine and glutamine or oxidation of methionine or tryptophan can induce structural changes in the overall protein structure, potentially leading also to changes in physical stability^1,9,10^. Both chemical and physical degradation might compromise biological activity and safety of the biologic^11^.

In early technical development of biologics, the risk-based predictive stability (RBPS), i.e. the prediction of stability behaviour and the practice of extrapolation to support development milestones, is a company’s internal decision. During the clinical development phase of biologics (and within clinical trial applications) limited extrapolation is used and accepted by health authorities (HA) worldwide^12,13^. Differently, in marketing authorisation applications and in the commercial phase, extrapolation is not accepted for biologics^14^. In the majority of cases, for clinical trial submissions, long-term stability testing is also performed and included in the submissions although some companies report using reduced protocols with fewer time points and conditions^15,16^. For small molecule drugs, the prediction of stability behaviour is based on accelerated stability data obtained at different temperatures and humidity levels. Experimental data are described by a kinetic model and expanded Arrhenius equation which allows prediction of long-term stability. Such predictions are for small drugs usually performed in accelerated stability assessment program^17–19^. However, the prediction of stability of biologics is still very limited and calculated by a classical approach using linear extrapolation. In clinical trial applications, regulatory expectations for risk-based predictive stability of biologics allow extrapolation for the same period as the available experimental data but not for more than 12 months^12^. Introducing RBPS into the development of biologics would have similar advantages as in small molecules. In particular, extensive time saving by performing stability studies at higher temperatures and predicting stability at the intended storage temperature. This approach allows also shelf-life estimation, quick evaluation of formulation and primary packaging, evaluation of comparability for DP or DS process changes, evaluation of stability for a wide range of storage conditions and more^15^. In addition, temperature excursions during storage or transport can be evaluated^20^.

It is considered that long-term stability predictions from accelerated stability data are usually not possible for biologics due to the complexity of temperature, time and concentration-dependent modifications. When studying aggregation, researchers either found non-Arrhenius behaviours^3–6^ or Arrhenius behaviour was very limited to narrow temperature and concentration intervals^7,8^. However, recent studies indicated the possibility to predict long-term aggregation profiles from accelerated stability data and some selected biophysical parameters by non-linear models, i.e. artificial neural networks^21^ or other multivariate approaches^21,22^. Arrhenius behaviour was also demonstrated for vaccine potency to be utilised to manage stability after a process change^23^, and to accurately predict long-term stability^20^. Long-term profiles of vaccine antigenicity that were predicted by Arrhenius kinetic analysis of six months of accelerated stability data were verified with up to three years of stability experimental data^20^.

In this article, we demonstrate accurate long-term (up to 3 years) stability prediction of multiple quality attributes for several therapeutic monoclonal antibodies and fusion proteins from short-term (up to 6 months) accelerated stability data combined by applying kinetic modelling using the Arrhenius equation. Specifically, we tested stability indicating quality attributes like purity, potency, aggregation, fragmentation and charge profile for five monoclonal antibodies and one fusion protein. Prediction of three years stability profiles at intendent storage condition was verified by experimental data: 96% of experimental data that were not used for building the model, lie within the calculated prediction intervals that were approximately three times narrower than the width of generic shelf-life specifications at the three year time point. More generally, this work is aimed to underlay the relevance and applicability of using a combination of short-term accelerated stability studies and kinetic modelling (beyond the currently used linear extrapolation) in the development of therapeutic monoclonal antibodies. This is of major interest to the biopharmaceutical industry and for general society as it enables shorter development periods of novel advanced biologics.

## Materials and methods

### Materials

All tested protein solutions were manufactured at Lek d.d. (member of Novartis), Mengeš, Slovenia and include IgG1 molecules adalimumab and rituximab, IgG2 molecule denosumab and mAb1 (IgG1) and mAb2 (IgG2) molecules in development and fusion protein etanercept. Stability studies on rituximab were done at protein concentration of 10 mg/mL. Protein was formulated in an innovator’s formulation, i.e. 0.7 mg/mL polysorbate 80.9 mg/mL sodium chloride and 5.25 mg/mL citric acid monohydrate at pH 6.5. Stability studies on adalimumab were done at protein concentration of 50 mg/ml. Protein was formulated in design around formulation comprised of 3.36 mg/mL adipic acid, 0.25 mg/mL citric acid monohydrate, 12 mg/mL mannitol, 1 mg/mL polysorbate 80 and 6.16 mg/mL sodium chloride at pH 5.2. Stability studies on denosumab were done at 70 mg/mL. Protein was formulated in an innovator’s formulation, i.e. 1.08 mg/mL acetic acid, 46 mg/mL sorbitol, 1 mg/mL PS20 at pH = 5.2. Stability studies on etanercept were done at 50 mg/mL. Protein was formulated in design around formulation comprised of 0.786 mg/mL citric acid, 13.52 mg/mL sodium citrate, 1.5 mg/mL sodium chloride, 10 mg/mL sucrose and 4.6 mg/mL lysine at pH 6.3. These four formulations are Novartis’ final drug product formulations identified as the most appropriate during the early drug product development. Stability predictions for this four proteins were made also for different batches of the same molecule where the batch represents a protein with uniform character and quality, within specified limits, and is produced according to a single manufacturing order during the same cycle of manufacture. Variability between batches originates from the production process. Stability studies on proteins in development mAb1 and mAb2 were done at protein concentrations ~ 5 mg/mL (mAb1) or ~ 150 mg/mL (mAb2). Proteins were formulated in different formulations and pH values between 5.5. and 7.0. All formulations were filled in different sizes of type I glass vials.

Chemicals for formulation preparation were of pharmaceutical grade, while chemicals and reagents for chromatography and electrophoresis were of HPLC grade.

### Analytical methods

The development of liquid dosage forms of biologics is mainly based on stability studies that are performed at accelerated ((25 ± 2) °C/(60 ± 5)% r.h.) and stress temperature ((40 ± 2) °C/(75 ± 5)% r.h.) whereas the intended commercial storage temperature is between 2 and 8 °C (we will use 5 °C throughout the article for simplicity reasons) with shelf-life usually between 2 and 3 years. Stability studies are performed at 25 °C and 40 °C in order to speed up changes in the quality attributes of the protein that can be detected by state-of-the-art analytical methods and save time spent for development. According to ICH guidelines stability indicating quality attributes of the protein and analytical methods are defined by force degradation studies that are designed in a way to identify likely degradation mechanisms^24^. As such, the intrinsic stability of the molecule is determined and the analytical methods indicating stability are validated. When developing a drug product for biologics, modifications/changes of different quality attributes are assessed. They can be divided into two groups: concentration-independent and concentration-dependent. The most prominent concentration-independent modifications are chemical modifications such as oxidation, (de)amidation, isomerisation, etc. These modifications are usually detected by peptide mapping (PepMap), imaged capillary isoelectric focusing (iCIEF), capillary zone electrophoresis (CZE), cation exchange chromatography (CEX) and reverse phase chromatography (RPC). The second type of concentration-independent modification is fragmentation that is detected by SDS capillary electrophoresis (CE-SDS). Concentration-dependent modifications are aggregation monitored by size exclusion chromatography (SEC) and formation of subvisible particles monitored by light obscuration (LO) or micro flow imaging (MFI) and formation of visible particles detected by visual inspection. Bioactivity, which is typically monitored by cell-based assays and by functional tests like surface plasmon resonance (SPR) or bio-layer interferometry (BLI), is impacted by the combination of the above-mentioned modifications. Frequently, purity is reported in addition. It is determined as the amount of the main peak assessed by iCIEF, CZE, CEX, CE-SDS or SEC.

### Size exclusion chromatography (SEC)

SEC analysis was performed on Agilent 1260 HPLC or Acquity H-class UPLC instrument using Waters Acquity BEH200 SEC, 1.7 µm, 4.6 mm × 150 mm column or similar. Mobile phase was 50 mM Na_2_HPO_4_/NaH_2_PO_4_ and 400 mM NaClO_4_ at pH 6.0 or 150 mM KH_2_PO_4_/K_2_HPO_4_ at pH 6.5. Monomer and aggregate peaks were detected by measuring absorbance at 210 nm. Chromatograms were analysed using Empower or Chromeleon software. Size of aggregates was determined from a calibration curve obtained from analysis of molecules of known molecular weights: thyroglobulin (669 kDa), IgG (150 kDa) and holo-transferrin (80 kDa). Accuracy of the measured amount of aggregates is between 0.05 and 0.1% while the amount of main peak (purity) is between 0.1 and 0.2%.

### Cation exchange chromatography (CEX)

CEX analysis was performed on Agilent 1260 HPLC instrument usually using MabPac SCX-10, 10 µm, 4 mm × 250 mm column from Thermo Scientific. Mobile phase A was 5.8 mM MOPSO, 4.4 mM Bicine, 12.2 mM CAPSO, 0.6 mM CAPS, pH 6.6, while mobile phase B was 12 mM MOPSO, 4 mM Bicine, 1.2 mM CAPSO, 5.8 mM CAPS, pH 11.5. Percentage of mobile phase B was increased from 15.9% to 63.7% in 26 min. Alternatively, MabPac SCX-10, 3 µm, 4 mm × 50 mm column from Thermo Scientific was also used in combination with mobile phase A composed of 5 mM TAPS, 5 mM CAPSO, 10 mM NaCl at pH 7.8 and mobile phase B composed of 5 mM TAPS, 5 mM CAPSO, 10 mM NaCl at pH 10.2. Percentage of mobile phase B was increased from 0 to 55 and 55 to 90% in 1 and 27 min, respectively. Absorbance chromatograms were analysed at 280 nm using Empower or Chromeleon software. Accuracy of measured amount of acidic, basic variants and main peak (purity) is between 0.5 and 1.5%.

### SDS capillary electrophoresis (CE-SDS)

CE-SDS analysis was performed by PA 800 Plus instrument from Sciex. Typically, bare fused-silica capillary with 50 μm of internal diameter and 20 cm long was used for the separation. SDS gel and SDS sample buffers were obtained from Sciex. Non-reducing conditions were always used. Protein-SDS complex was alkylated with iodoacetamide or *N*-ethylmaleimide in a 45–70° C water bath for 10 min. After samples were cooled, they were electrokinetically introduced into the capillary and separated by applying constant voltage. Absorbance electropherograms were analysed at 210 nm using Empower or Karat software. Accuracy of measured amount of fragments is between 0.2 and 0.3% while for the amount of main peak (purity) is between 0.3 and 0.5%.

### Bioactivity

In the complement-dependent cytotoxicity (CDC) test^25^, Raji B cells (isolated from a patient with Burkitt’s lymphoma, from American Type Culture Collection (ATCC)) are incubated with different protein concentrations and a fixed concentration of rabbit complement. Concentration-dependent killing of the Raji cells is analysed after 2 h where viability of the cells is measured with indirect determination of ATP concentration (by measuring luminescence produced by the ATP-consuming luciferin-luciferase system). In the case of tumor necrosis factor-alpha (TNFα) antagonists such as etanercept and adalimumab reporter gene assay was used as descibed in ^26^. Activity of the protein’s test sample is determined by comparison to a reference standard. The samples and the standard are normalised on the basis of protein content. Relative potency is then calculated using a parallel line assay according to the European Pharmacopoeia. Accuracy of measured relative potency is around 10%.

### Kinetic model

**Figure 1:**
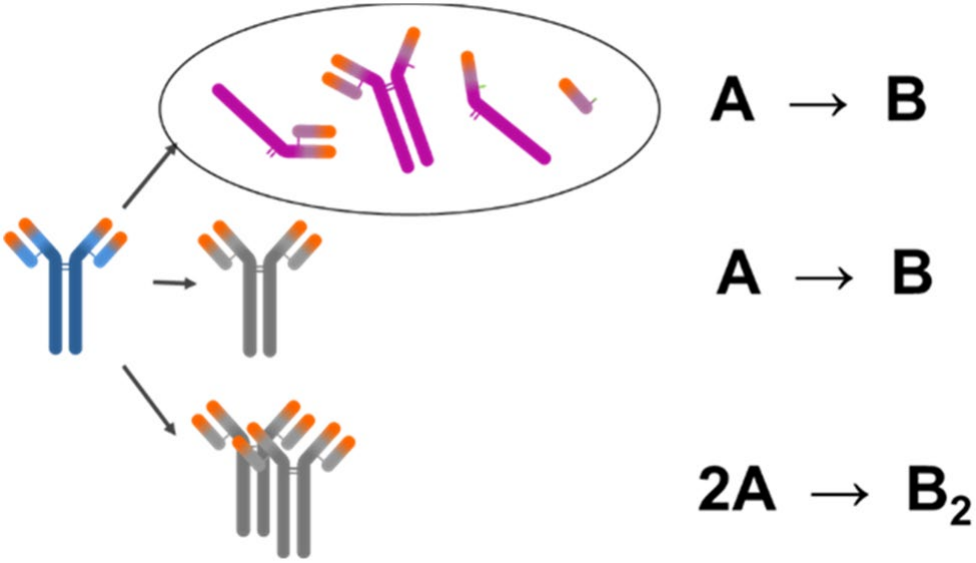
Simplified scheme of protein degradation and aggregation pathways. Top scheme: fragmentation, middle scheme: chemical modifications, bottom scheme: aggregation. Figure made in PowerPoint 2016 (Microsoft, Redmond, WA, USA).

Kinetic models of protein processes are composed from dynamic rate terms of quality attributes and the empirical Arrhenius equation^27^ that describes the temperature dependence of rate constant. Quality attributes considered are purity and sum of impurities, i.e. fragments, charge variants and aggregates. When studying stability of the drug product from the fragmentation standpoint, we are usually interested in the sum of fragments, meaning that the apparent chemical reaction can be approximated as A → B (see Fig. 1, top scheme). The same reaction scheme was applied also when assessing chemical modifications (middle scheme). We can monitor single amino acid residue modification with PepMap or the sum of acid or basic variants by iCIEF, CZE or CEX. The latter approximation was reported to have been successfully used for describing chemical modification of a single amino acid residue^28–30^. In the examined cases, aggregation was monitored by the SEC method and the sum of aggregates was determined. Dimers were the most abundant species among the aggregates for all tested proteins at all time points. Thus, the fastest pathway was formation of dimers, especially at *T* < 40 °C, where only minor amounts of larger aggregates were detected. Chromatogram overlays of mAb1, which show total area under the curve did not change with time even at 55 °C storage temperature, is provided in Figure S1, while the same was observed for other mAbs for all conditions tested as well. This finding for examined monoclonal antibodies largely simplifies the model for aggregation, which can be described by the chemical reaction 2A → B_2_ (bottom scheme in Fig. 1). We are aware that aggregation pathways can be more complex^31–33^ and can vary depending on temperature, time, protein type, concentration, formulation, process and more^3,34^. Depending on the conditions, we may observe native or non-native aggregation that proceeds via different reactive intermediates towards different oligomers (dimers, trimers, etc.), soluble polymers and precipitates^35^. Nevertheless, if aggregation conditions are carefully chosen, the process can be successfully described using the simple model mentioned.

In our kinetic modelling, we assume apparent one-step irreversible chemical reactions A → B and 2A → B_2_ which can be described by equations:

1a $$\frac{d[\mathrm{A}]}{dt}=k(T){[\mathrm{A}]}^{n}$$

1b $$\frac{d[{\mathrm{B}}_{i}]}{dt}=k{(T)[\mathrm{A}]}^{n}$$

Equation (1a) describes how concentration of A changes over time *t* and Eq. (1b) how concentration of B_i_ (i = 1 or 2, respectively) changes over time *t*, both with respect to reaction order *n*, temperature-dependent rate constant *k*(*T*). A → B and 2A → B_2_ reactions are first (*n* = 1) and second (*n* = 2) order, respectively. Importantly, these chemical reactions should be understood as ‘apparent’ because they do not necessarily correspond to the actual dynamical processes in our protein systems, i.e., these apparent reactions should be understood as effective phenomenological dynamics observed in experiments.

In order to predict stability at different temperatures (in the temperature range from 5 to 60 °C, as relevant for our monoclonal antibodies), we use the Arrhenius equation^27^ that empirically describes temperature dependence of rate constant, *k*(*T*)

2 $$k\left(T\right)={A}_{f} {\mathrm{e}}^{-\frac{{E}_{a}}{RT}}$$

where *A*_*f*_ represents the frequency factor and *E*_a_ the activation energy. In this case *k*(*T*) represents the degradation rate constants that applies to a particular quality attribute of the molecule, such as aggregation, fragmentation, chemical modifications and bioactivity. Based on the assumption explained below we approximated all kinetic processes as first order chemical reactions. The combination of Eqs. (1a) or (1b) and (2) thus results in simple first order degradation kinetic model, namely for purity quality attributes:

3 $$\left[\mathrm{A}\right]\left(t,T\right)=[{\mathrm{A}}_{0}]{e}^{-k\left(T\right)t}$$

and for the (sum of) impurities by taking into account that [A] + [B] is total concentration:

4 $$[\mathrm{B}]\left(t,T\right)={1-(1-[\mathrm{B}}_{0}]){e}^{-k\left(T\right)t}$$

We should comment that we use first order degradation kinetic model to describe degradation and aggregation processes for the following reasons: We use temperature conditions and time intervals (i) where only one degradation/aggregation pathway dominates^34^, (ii) where relatively small changes in the quality attributes of the protein are detected, usually up to 20% in 3 months at 40 °C, (iii) where the amount of formed higher-order aggregates not detected by SEC is negligible^33^, (v) where only aggregates detected by SEC, among which dimers are the most abundant, are considered, thus more complex mechanisms of nucleation and growth can be omitted^3^, and (iv) aggregation is studied at constant protein concentration, therefore second order kinetics can be approximated by first order degradation kinetic model.

### Kinetic model fitting and calculation of prediction intervals

Kinetic model parameters are calculated using data from the accelerated stability study for a single batch. The set of experimental stability data for a given batch of biologics thus consists of time interval (up to 6 months), temperature conditions, and analytical measurements of sample quality profile, including bioactivity.

Using this data we simultaneously fit a single model, not only in time, but also for different temperatures (using Arrhenius relation), which crucially contributes to the robustness of the fitting. Precisely this multi-parameter fitting allows for reliable and robust extrapolation in time (and also in temperature) using the fitted kinetic model, well beyond the measured range of the parameter space. A single analytical measurement of sample pulled at *t* = 0 was initial experimental value for all different temperatures datasets.

The kinetic model is fitted to a given set of experimental stability data numerically, using non-linear least-squares optimisation, which minimises the sum of squared residuals *SS*_res_:

5 $${SS}_{res}={\sum }_{i}{({y}_{i}-{p}_{i})}^{2}$$

where *y*_i_ are the actual measurements and *p*_i_ the calculated model points. Model parameter fitting was done in Python using the SciPy library’s curve_fit function set to the “dogbox” method^36^. To improve the numerical stability, we used a different parametrization of Eq. (2), solving the problem for parameters *E*_a_ and *k*(*T*_ref_):

6 $$k\left(T\right)=k({T}_{ref}) {e}^{-\frac{{E}_{a}}{R}(\frac{1}{T}-\frac{1}{Tref})}$$

We chose the intended storage temperature (5 °C) as ${T}_{ref}$, so the solution of the problem directly provides the rate of degradation at this condition, while the rate at other temperatures can be computed using the model. To verify the quality of the model fit and compare it across different data sets we used the coefficient of determination (*R*^2^), which is a normalised score with the maximum value of 1, achieved when the model fits the experimental data perfectly:

7 $${R}^{2}=1-\frac{{SS}_{res}}{{SS}_{tot}}$$

where ${SS}_{tot}$ is defined as:

8 $${SS}_{tot}={\sum }_{i}{({y}_{i}-\overline{y})}^{2}$$

To further enhance and, more importantly, evaluate the quality of our extrapolation, we use Monte-Carlo simulations to construct prediction intervals for the kinetic model. From a given set of experimental data we generate an additional 399 sets of perturbed data, where we add random variation (noise) to the original measurements sampled from a Gaussian distribution with zero mean and standard deviation set to the measuring equipment’s specified error (equal to method accuracy noted in the description of each analytical method). We repeat the parameter estimation and prediction for the desired conditions for each perturbed set to obtain a distribution of predictions. The 2.5th and 97.5th percentiles give us the 95% probability confidence interval, while the 95% probability prediction interval additionally accounts for the dispersion due to measurement errors and is computed as in ^37^. The upper and lower bounds are computed separately as the interval can be asymmetric. The described regression and extrapolation are established approaches when dealing with fitting the non-linear model functions to experimental data^38,39^. The calculations were implemented as a custom widget within the Orange data mining framework (https://orangedatamining.com/)^40^.

### Linear regression

According to ICH guidelines^41^, linear regression is applied for modelling of stability profiles and for shelf-life calculations. Linear extrapolations are accepted as well by health authorities to support clinical development phase of drugs^12^. In our work, linear extrapolation is used to allow for comparison with our improved kinetic modelling. The coefficients, intercept and slope, of the linear model were calculated by using measured levels of quality attributes at given pull points for samples stored at intended storage condition (5 °C). Coefficients and 95% prediction intervals were calculated by applying *lm* and *predict* functions from the *stats* library of R^42^.

### Verification of model extrapolation against experiments

As the key final verification of our in-time extrapolation (i.e. prediction) of the changes of quality attributes, we compare the calculated prediction intervals with actual full measured stability data (over 3 years) that is available at time of writing. Verification is calculated/denoted as number of points lying within the 95% probability prediction interval among all experimental stability data points. Short-term accelerated or intended condition stability data (of 6 months) used in model fitting are excluded from this verification pool of data. The verification of model extrapolation against long-term experimental stability data is used here to prove the correctness of our methodology; however, the idea is precisely that these long-term experimental stability studies may not be needed anymore, if using more advanced kinetic modelling beyond linear extrapolation.

## Results

### Long-term stability prediction from accelerated stability data

**Figure 2:**
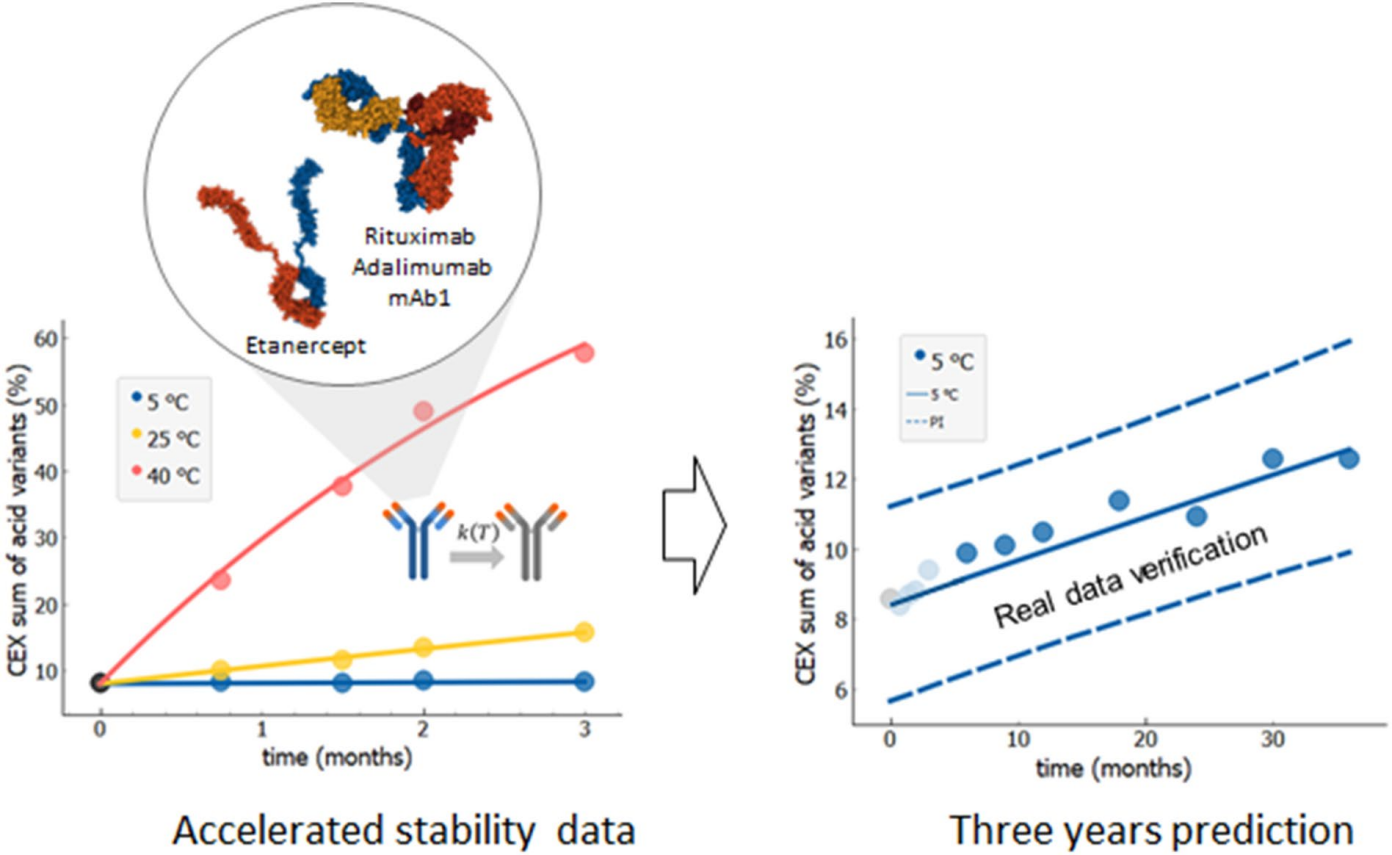
Long-term (36 months) stability prediction based on accelerated stability data (3 months) and kinetic modelling which included Arrhenius temperature dependence of kinetic rates. Accelerated stability data (left, data points) are used to develop the kinetic model (left, full lines) to predict long-term stability at intendent storage conditions (right). The 95% probability prediction interval designated by dashed blue lines is verified by long-term experimental data not used in model fitting (right, dark blue data points). Stability prediction was evaluated for three mAbs (rituximab, adalimumab and mAb1) and for one fusion protein etanercept. Shown data points are values of sum of acid variants obtained by CEX, one of the stability indicating quality attribute, measured for one batch of rituximab. Each point represents single measurement (*N* = 1). Measurement accuracy SD = 1.5% was estimated by several measurements of control samples over multiple analytical runs. Protein structures created using 1HZH^43^, 1H3W^44^ and 3ALQ^45^ structures from RCSB PDB (rcsb.org)^46^ using Mol*^47^ and Snagit (TechSmith, Okemos, MI, USA).

Figure 2 shows the exemplary long-term (36 months) stability prediction based on accelerated stability data (3 months) and kinetic modelling using the Arrhenius temperature dependence of kinetic rates. Specifically, the image shows results for rituximab molecule and the prediction for increase of the sum of acidic variants. We used experimental data for 3 months (Fig. 2, left, data points) and for three different temperatures (5, 25 and 40 °C), which were obtained within an accelerated stability study. These experimental data were then fitted with the kinetic model by using Eqs. (4) and (6) (Fig. 2, left, full lines). We fitted simultaneously all three measured temperatures. The 3-year prediction curve for the change of selected quality attribute in time was determined, together with the 95% probability prediction interval (Fig. 2, right, full and dashed lines, respectively). Finally, the prediction interval obtained from the 3 months accelerated stability data is validated against the long-term experimental data (Fig. 2 right, dark blue data points), showing excellent agreement between the prediction and actual measurements, with all measured data points within the prediction interval and very close to the prediction curve.

### Prediction of changes of multiple stability quality attributes

**Figure 3:**
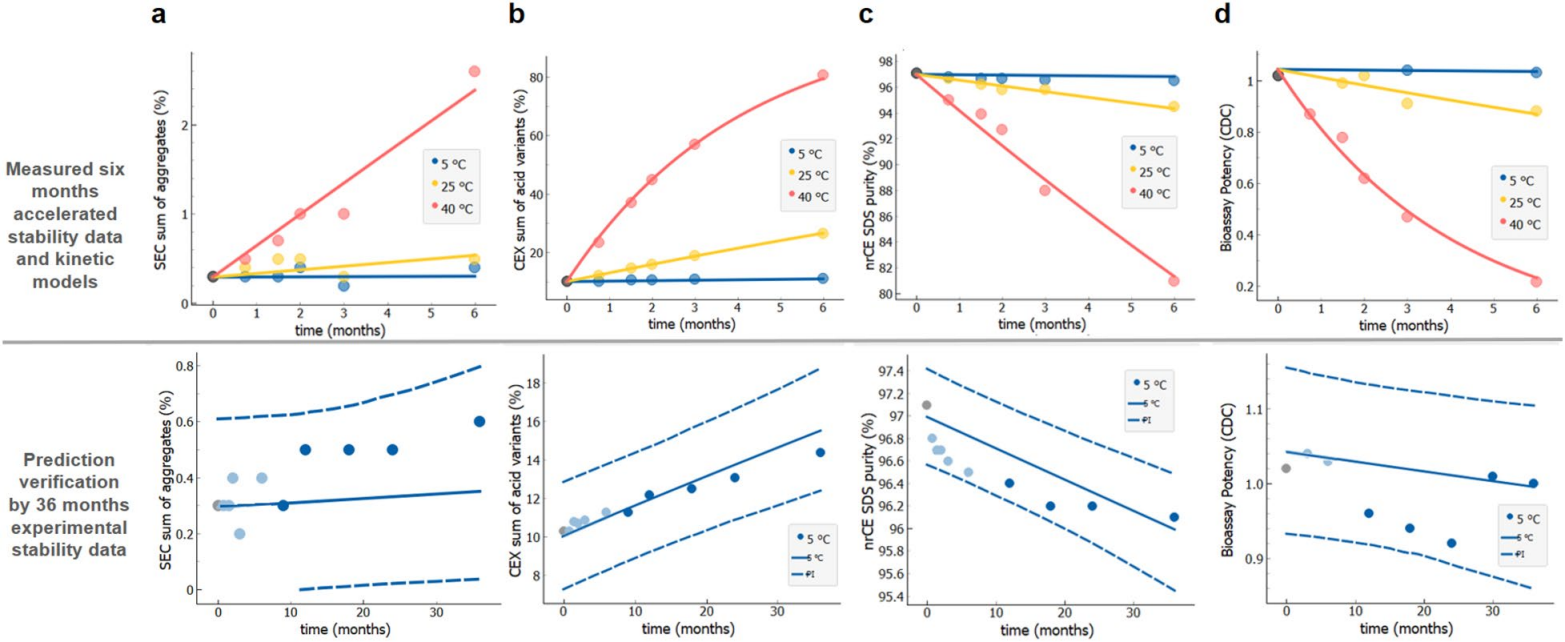
Long-term stability prediction of different stability indicating quality attributes for rituximab. **(a)** Sum of aggregates by SEC (*R*^2^ = 0.92), **(b)** sum of acidic variants by CEX (*R*^2^ = 1.0), **(c)** purity by nrCE-SDS (*R*^2^ = 0.99), **(d)** complement-dependent cytotoxicity activity (CDC) (*R*^2^ = 0.99). Accelerated 6 months experimental stability data (upper panels, data points) are used for model parameter calculations (upper panels, full lines). Prediction intervals (dashed blue lines in lower panels) are validated against the available long-term experimental data (bottom panel, dark blue data points). All experimental data are from the single material batch. Measured value at *t* = 0 is designated by a black solid circle.

Figure 3 (upper panels, data points) show measured data from (accelerated) six months stability study of the key stability indicating quality attributes of rituximab (same batch as above). These accelerated stability data were analysed—i.e. fitted and extrapolated separately for each stability quality attribute: sum of aggregates by SEC (Fig. 3a), sum of acidic variants by CEX (Fig. 3b), purity by nrCE-SDS (Fig. 3c), and complement-dependent cytotoxicity activity CDC since this is the main mode of action of rituximab. (Fig. 3d). The obtained stability profiles and prediction intervals for long-term 36 month period (intended drug shelf-life) at intended conditions (5 °C) are shown on the lower panels of Fig. 3 (full and dashed lines). Similarly as above, the predictions are validated against long-term experimental stability data (Fig. 3, bottom panel, dark blue data points not used for model building), showing excellent agreement with the predictions based on 6 months accelerated stability data. Furthermore, we verified whether first order degradation kinetic model can be successfully applied to predict long-term stability profiles of sum of aggregates for adalimumab, etanercept, denosumab and mAb1. As shown on Figure S2, 95% probability prediction intervals encapsulate all experimental long-term stability data.

### Improving robustness of kinetic model from accelerated data by goodness of fit and by temperature sub-sampling

We assess the relevance and appropriateness of the chosen kinetic model using only accelerated stability data. The most straightforward criterion that measures the appropriateness of the applied kinetic model is the goodness of fit (*R*^2^) value as introduced in the Methods section. In the presented study we achieved 0.9 < *R*^2^ < 1 in most cases, indicating good quality of fits. Note that observing low *R*^*2*^ indicates either high values of measurement errors in the stability data or improper selection of the kinetic model.

The second criterion of model robustness is based on sub-sampling of the experimental accelerated stability data, by joint fitting data from fewer selected temperatures. Typically, our accelerated stress stability studies are performed at least at three different temperatures (e.g. see Fig. 3), and model fitting is performed with all accessible short-term data (i.e. with all temperatures). However, to test the robustness, we include only subsets of data (i.e. from fewer temperatures, minimal three temperatures) in fits and observe the obtained fit parameters *E*_a_ and *k*(*T*_ref_). If fitting results in the same model parameters for different subsets and the full set of data (all temperatures), this indicates that the kinetic model is robust in the whole temperature range. For example, if stability data is available for 5 °C, 25 °C, 40 °C and 50 °C temperatures one can get 5 different data subsets, where minimally three different temperatures are included in a single data subset: {5 °C, 25 °C, 40 °C}, {5 °C, 25 °C, 50 °C}, {5 °C, 40 °C, 50 °C},{ 25 °C, 40 °C, 50 °C} and {5 °C, 25 °C, 40 °C, 50 °C}.

**Figure 4:**
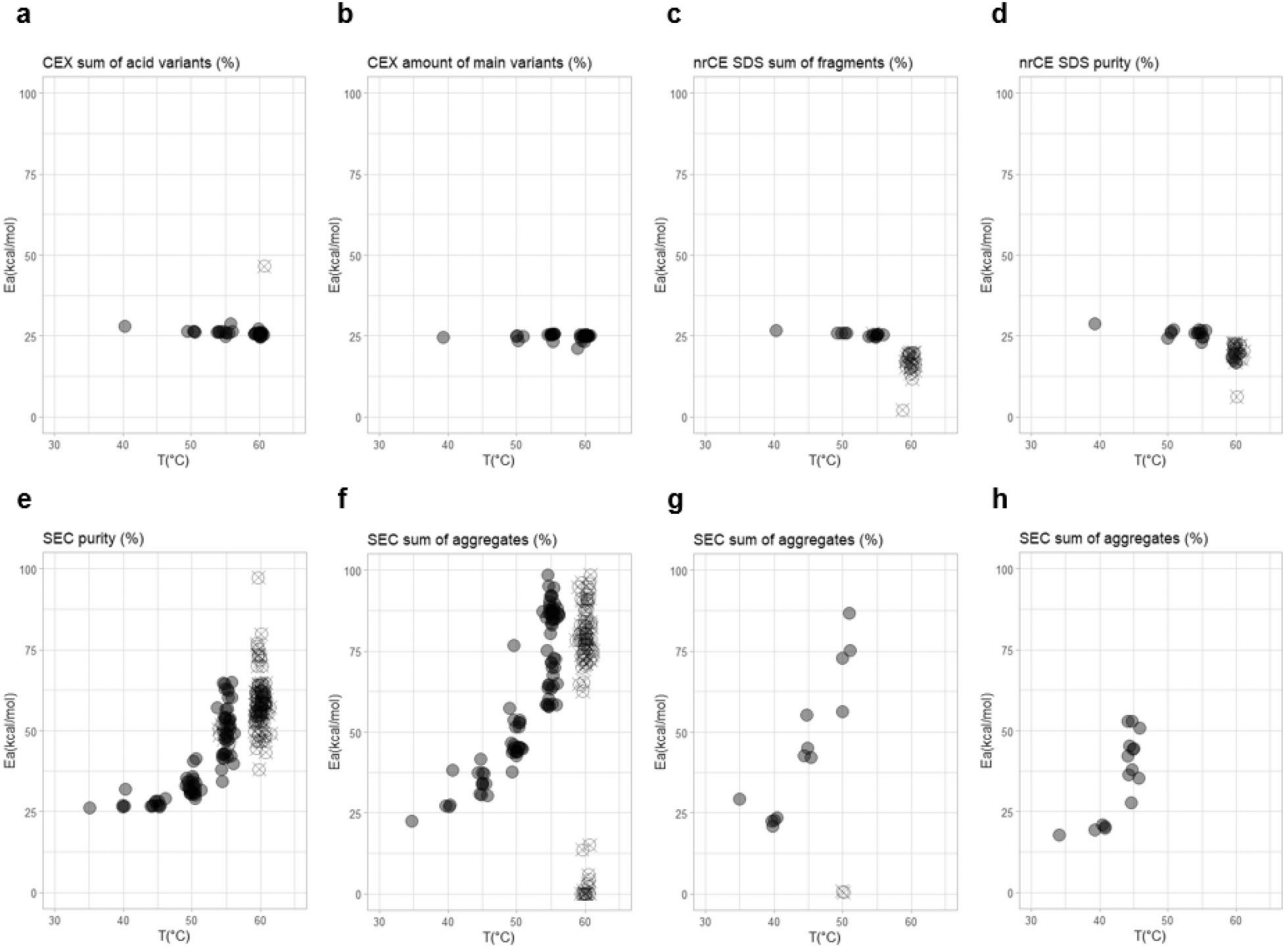
Robustness of kinetic model by the analysis of temperature sub-sampling of accelerated stability data. Each circle represents calculated *E*_a_ (on y-axis) and the highest temperature (on x-axis) for one subset. Each subset contains at least 3 different temperatures. Parameters *E*_a_ are determined for all combinations of subsets for different quality attributes of one batch of mAb1 molecule **(a–f)** and for sum of aggregates for one batch of etanercept **(g)** and mAb2 **(h)**. Goodness of fit for each determined activation energy is colour-coded by the colour of the data points circles; grey circles indicate (*R*^2^ ≥ 0.9); crossed empty circles indicate *R*^2^ < 0.9.

The described approach was confirmed by using mAb1 molecule stability data in temperature range up to 60 °C. Figure 4 shows activation energies as obtained from first order kinetic model fitting of different temperature data subsets. For charge variant analysis (Fig. 4a,b) and for fragmentation analysis (Fig. 4c,d) we have data available for six storage conditions (5 °C, 25 °C, 40 °C, 50 °C, 55 °C and 60 °C) resulting in 42 different combinations with at least three different temperatures in a subset. For sum of aggregates analysis (Fig. 4, panels e and f) where additionally data for 35 °C and 45 °C temperatures are available, the number of combinations is 219. The analysis of charge variants (Fig. 4a,b) resulted in very similar activation energy *E*_a_ values (approximately 25 kcal/mol) for all temperature sub-sampling combinations, including 60 °C condition. This clearly demonstrates that first order degradation kinetic model for chemical modifications follows the Arrhenius relation across the whole temperature range (5–60 °C) in the event of mAb1 molecule. In the fragmentation analysis (Fig. 4c,d), *E*_a_ of 25 kcal/mol is obtained for all temperature combinations, except for subsets including 60 °C temperature. Expectedly, in these subsets *R*^2^ was below the threshold level of 0.9. Collectively these two findings suggest that at higher temperatures additional mechanisms of fragmentation are present, which are beyond the dynamics covered in our (rather simple) kinetic model. The aggregation process (Fig. 4e,f) is even more complex involving additional aggregation mechanisms already at *T* > 45 °C, reflected by increasing *E*_a_ above this temperature. Activation energy *E*_a_ for purity parameter (Fig. 4e) and sum of aggregates (Fig. 4f) is around 25 kcal/mol, the same as in case of chemical degradation and fragmentation. Note that on Fig. 4, panels g and h, model verification results for aggregation of fusion protein etanercept and mAb2 are shown. For etanercept and mAb2 *E*_a_ is around 25 kcal/mol and 20 kcal/mol, respectively, when we include data subsets up to 40 °C. When we include data subsets above 40 °C, obtained *E*_a_ increases for both proteins, suggesting the change of aggregation kinetics mechanism.

### Verification of stability prediction using long-term stability data

Long-term stability data is the most powerful way for verification of the stability prediction approach. However, if full long-term experimental data are available, the question “What is the value of accelerated experiments and modelling-based predictions?” naturally occurs. In the context of our work, we resorted to the verification with long-term stability data only to convince the reader about the success and robustness of the accelerated stability data based prediction approach and, moreover, a proof that a range of stability affecting processes in multiple therapeutic monoclonal antibodies follow apparent first order degradation kinetic model that can be applied for prediction beyond the measured data. Table 1 shows a summary of prediction accuracy for different stability indicating quality attributes (purity, potency (CDC for rituximab and reporter gene assay for adalimumab and etanercept), aggregation, fragmentation and charge profile) of rituximab, adalimumab, etanercept and mAb1. Note that mAb1 molecule is still in early development phase with only limited number of samples characterized by potency bioassay. Hence no potency could be predicted yet. Other quality attributes such as subvisible particles, degree of glycosylation, content, turbidity, color, pH etc. are usually not stability indicating for mAbs and are therefore not evaluated. The prediction accuracy is assessed by the percentage of long-term experimentally measured data points that fall within model-based prediction intervals. We utilised the same analysis as in Fig. 3, except that for each molecule several batches (technical replicates) were analysed. Each batch was evaluated independently. First, for each batch and each quality attribute first order degradation kinetic model was built by using a six months stability dataset including 5 °C, 25 °C and 40 °C data points. The second step, prior the long-term prediction, was datasets clean-up. It turned out that among 398 built kinetic models there were few models with poor alignment between measured 6 months stability data and the model. In most cases the reasons for poor modelling were out-layers and/or high analytical variability of data sets with limited data points. As a threshold for acceptable datasets was goodness of fit R^2^ ≥ 0.9. In total 359 datasets/models passed into a third phase where each data point measured after the first 6 months was checked whether it fell within calculated 95% probability prediction intervals or not. Prediction accuracy for each quality attribute and molecule was then calculated for all batches as the percent of all data points that fell within the prediction intervals. Impressively, as clearly shown in Table 1, we achieved more than 90% accuracy for all molecules and all stability quality attributes assessed, showing exceptional reliability and robustness of our long-term stability prediction.

**Table 1:** Percent of batches where long-term experimental stability data are in agreement with long-term stability predictions.

	mAb1	Rituximab	Etanercept	Adalimumab
CEX amount of main variants (%)	100	91	100	99
CEX sum of acid variants (%)	100	97	100	99
nrCE SDS purity (%)	100	95	100	98
nrCE SDS sum of fragments (%)	100	No data	No data	No data
Relative potency (%)	n.a.	97	100	100
SEC purity (%)	100	98	90	100
SEC sum of aggregates (%)	100	94	90	97

Percent of batches that meets the criteria is obtained as the ratio of experimental long-term data points between 6 and 36 months at 5 °C that fall within the prediction interval relative to the total number of all measured values during that time. Prediction intervals are determined from the 6 months accelerated stability data . Only batches passing the criterion goodness of model fit *R*^2^ ≥ 0.9 are considered in the analysis. Numbers of passed batches are 8 (molecule in development mAb1), 36 (rituximab), 11 (etanercept) and 19 (adalimumab). “no data” indicates that no accelerated and long-term experimental stability studies were performed for that quality attribute and protein, “n.a.” indicates not applicable since not enough data have been collected.

The widths of model-based prediction intervals are generally narrower compared to the current state-of-the-art generic drug product specifications set by the guidelines for first in human studies of monoclonal antibodies (see e.g. ^48^). Calculations in Table 2 utilised the same data analysis as in Table 1 and show the average prediction interval widths in comparison to the specification widths defined by the generic specifications for first in human studies, and their quotient expressed in%. Generic specifications for potency, purity, aggregation and fragmentation are defined as absolute values in the guidelines as acceptance criteria for mAb drug substance and drug product^48^ ( relative potency not less than 50% and not more than 150%, SEC purity not less than 90.0%, SEC sum of aggregates not more than 5.0% and nrCE SDS purity not less than 90.0%). For acceptance criteria of nrCE SDS sum of fragments authors suggest to report result or not more than x.x % limit where in analogy to sum of aggregates a criteria was set to not more than 5.0% ).Generic specifications for charge variant profiles are molecule-dependent and thus there is no clear target values that can be broadly applied as platform acceptance criteria. In presented study acceptance criteria for charge variant are set for 10% wider as than the observed results as an option proposed by Kretsinger et al. ^48^. Namely, acceptance criterion for sum of acidic variants is “less than its initial value + 10%”, while acceptance criterion for amount of main variant is “more than its initial value -10%”. Initial values were calculated as an average of all batches of a molecule at pull point *t* = 0 and rounded to the nearest five. As shown in Table 2 calculated model-based prediction intervals at 36 month time point are substantially narrower compared to the generic specifications for all quality attributes and for all studied molecules. Despite the narrowness of the prediction intervals, we still achieved better than 90% accuracy of prediction of all stability quality attributes (Table 1).

**Table 2:** Determined prediction interval widths relative to the current state-of-the-art mAb platform specifications for drug products^48^.

	mAb1	Rituximab	Etanercept	Adalimumab
CEX amount of main variants (%)	30% (13.3/45)	16% (6.4/40)	23% (10.4/45)	32% (9.5/30)
CEX sum of acid variants (%)	24% (8.5/35)	32% (6.6/20)	25% (6.4/25)	33% (6.7/20)
nrCE SDS purity (%)	20% (2.0/10)	24% (2.4/10)	19% (1.9/10)	26% (2.6/10)
nrCE SDS sum of fragments (%)	25% (1.2/5)	No data	No data	No data
Relative potency (%)	n.a.	46%(46.0/100)	75%(75.0/100)	74% (74.0/100)
SEC purity (%)	40% (4.0/10)	38% (3.8/10)	24% (2.4/10)	47% (4.7/10)
SEC sum of aggregates (%)	18% (0.9/5)	14% (0.7/5)	11% (0.5/5)	17% (0.9/5)

Values in brackets are an average of width of calculated model-based prediction intervals divided by the width of the mAb platform specification. The widths of model-based prediction intervals are given at 36 months as obtained from fitting of 6 months short-term stability study data. These prediction interval widths were calculated from exactly the same data set as the prediction accuracies shown in Table 1.

### Prediction accuracy of kinetic modelling vs. linear extrapolation

**Figure 5:**
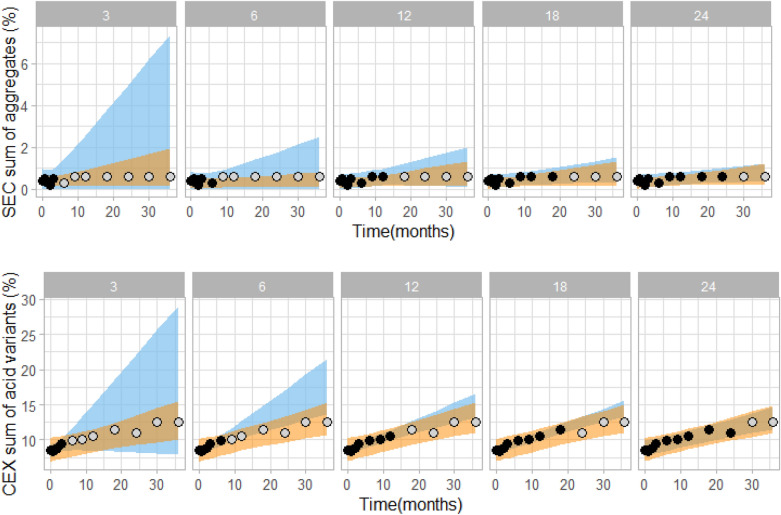
Comparison of kinetic model (orange) and linear extrapolation (blue) prediction intervals by using 3, 6, 12, 18 or 24 month experimental stability data (black data points). In grey, remaining measured data points at intended storage are shown for visual verification of the prediction intervals. Data shown is for one rituximab batch stored at 5 °C. For kinetic modelling data points from 25 and 40 °C conditions up to the respective time point were used (data points not shown for clarity reasons).

According to ICH guidelines^41^ stability profiles and shelf-life are determined by using linear regression models of measured data at intended conditions. To compare the kinetic modelling approach with the linear extrapolation we evaluated the same experimental data as above with the linear extrapolation approach. We compared accuracy of the prediction and prediction intervals for different extrapolated time periods. Specifically, we calculated prediction intervals with kinetic modelling and with linear extrapolation by using 3, 6, 12, 18 or 24 months experimental stability data (note: in analyses above only 6 month data was used for prediction calculations). Figure 5 evidently shows that kinetic modelling resulted in narrower prediction intervals compared to linear extrapolation. For example, by using kinetic modelling already after 6 months of stability study the sum of aggregates is predicted to be less than 1% at 36 month intendent condition time point, which is in agreement with the experimental long-term stability data. On the other hand, by using linear extrapolation a similar range can be predicted only after 24 months of stability study (Fig. 5, upper panel). Similarly, for the sum of acidic variants, good enough prediction intervals can be obtained by kinetic modelling 15 months earlier (after 3 months) compared to linear extrapolation (after 18 months, Fig. 5, lower panel). We defined a good enough prediction interval as prediction interval that falls within generic specifications interval. This analysis was performed on a single batch of rituximab. Additional examples demonstrating higher accuracy of kinetic modelling compared to liner regression are shown on Figure S3.

**Figure 6:**
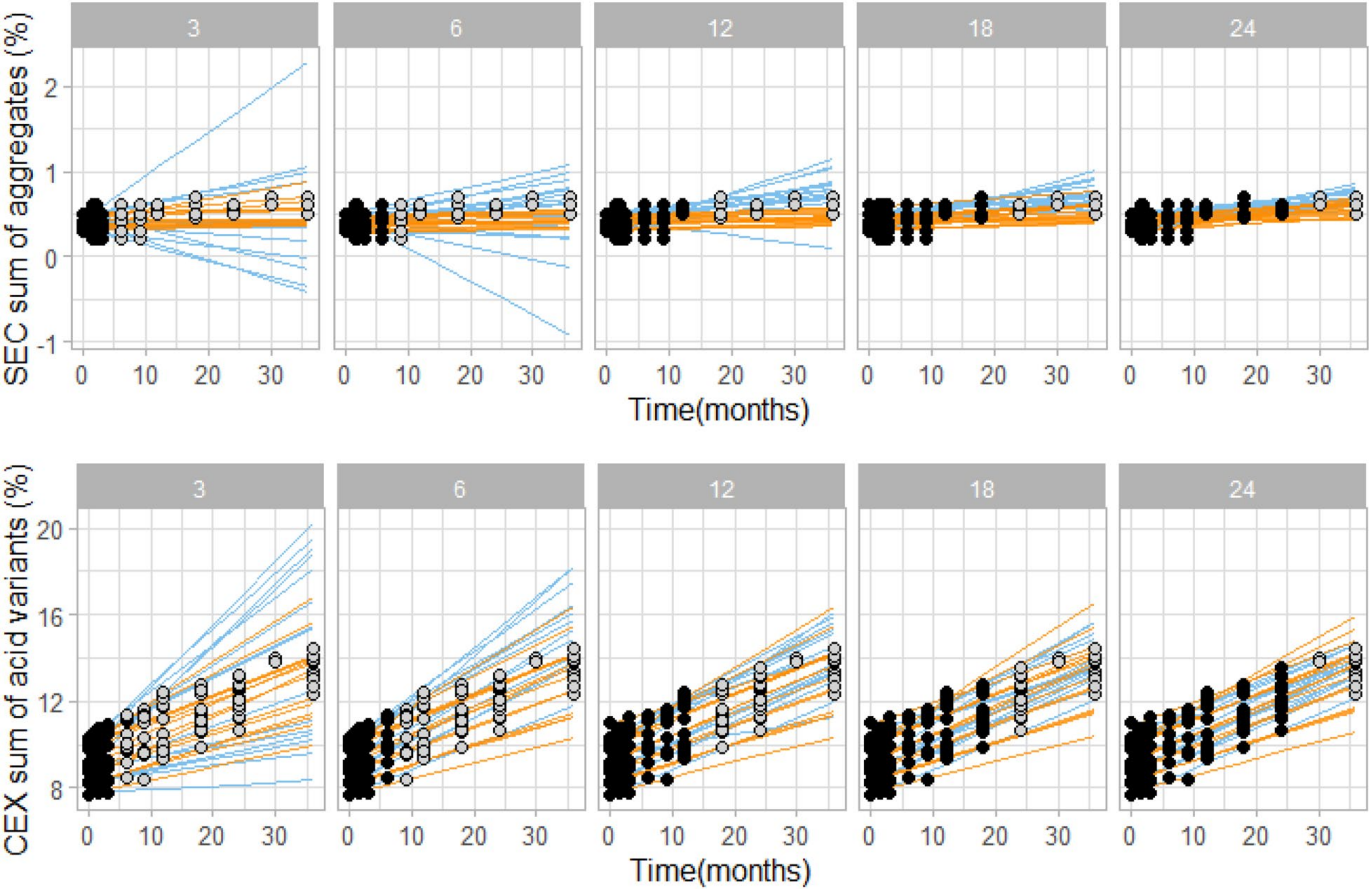
Comparison of kinetic modelling (orange) and linear (blue) extrapolation, showing prediction curves based on 3, 6, 12, 18 or 24 month experimental stability data (black data points). In grey, remaining measured data points at intended storage are shown for visual verification of the prediction intervals. Data shown is for 23 rituximab batches stored at 5 °C. For kinetic modelling data points from 25 and 40 °C conditions up to the respective time point were used (data points not shown for clarity reasons).

In Fig. 6 a comparison of kinetic modelling and linear extrapolation is shown for analysis of multiple (23) batches of rituximab, showing predicted curves (i.e. not prediction intervals as in Fig. 5) of sum of aggregates (Fig. 6, upper panels) and sum of acidic variants (Fig. 6, lower panels). Clearly, the kinetic model is able to more accurately predict the actual changes in the quality attributes. Especially when using only short-term data for model building linear extrapolation overestimates the actual variability of the system, while kinetic model-based predictions are accurate and do not vary between batches already when using 3 month data only. To sum up, result comparison of prediction intervals for a single batch (Fig. 5) together with result comparison of prediction curves for multiple batches provides a clear conclusions that kinetic modelling can more accurately predict the assessed stability quality attributes than linear extrapolation.

### Approaching rapid stability prediction by exploiting a month-long stability study at temperatures beyond traditional stress conditions

Higher temperatures lead to faster kinetics of degradation processes. This fundamental knowledge of chemistry could potentially be used for accelerating stability prediction even further. Two limitations, arising from the nature of the observed degradation, for using kinetic modelling can potentially affect such rapid screening: (i) the temperature up to which the simple kinetic model can sufficiently/successfully describe observed changes and (ii) the variability of the experimental data, which sets the minimal time interval in which data has to be collected for the model to give sufficiently robust fit. As discussed in Fig. 4, some stability quality attributes follow the same mechanisms at temperatures above 40 °C as at intendent storage conditions (5 °C) and in these cases degradation can be successfully described by (apparent) first order degradation kinetic model across whole temperature range. For presented charge variant studies, acceptable maximum storage temperature could be as high as 60 °C, for fragmentation 55 °C, etc. However, acceptable temperature ranges depend on the molecule and should be assessed on a case-by-case basis.

**Figure 7:**
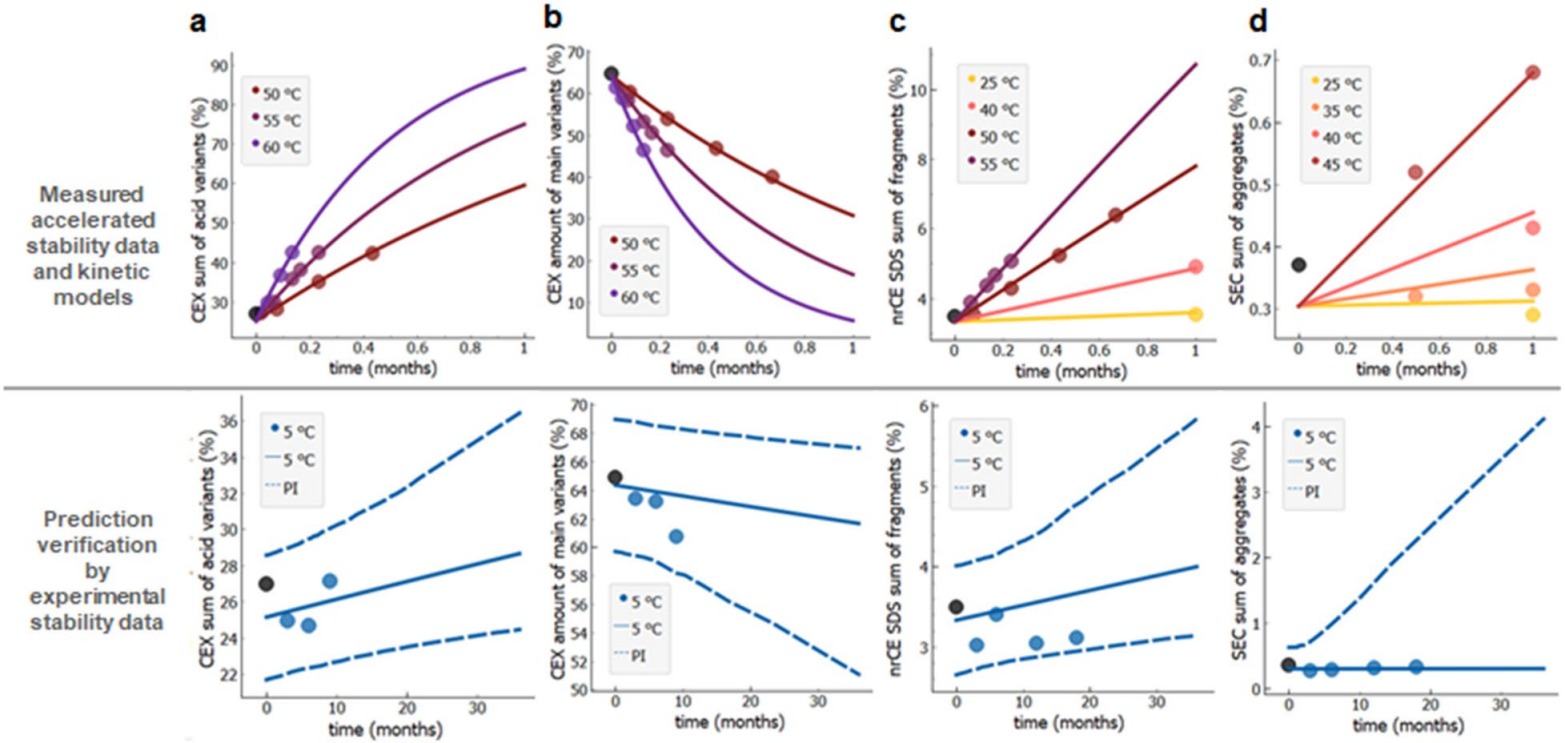
Rapid stability prediction for mAb1 molecule from stability data at temperatures 25 to 60 °C. **(a)** Two weeks data for acidic variants, **(b)** three weeks for main variants, both measured by CEX, **(c)** four weeks for fragmentation measured by nrCE-SDS and **(d)** four weeks for sum of aggregates by SEC. Highly accelerated experimental stability data (data points) and model fits (full lines) are shown in the upper panels, to generate prediction curves (full line) and prediction intervals (dashed lines) with measured data (solid circles) in the bottom panels. Note that different temperature intervals had to be used for different stability quality attributes, depending on the nature of degradation process kinetics. Measured value at *t* = 0 is designated by a black solid circle.

To achieve accurate and narrow prediction intervals in the shortest possible time, we generally obtain stability data from three or more temperature conditions, which are up to the temperature at which kinetic mechanism changes and the simple kinetic model cannot describe the observed change. How to identify this maximal temperature has been discussed above (under the Fig. 4). Here we assessed accuracy and precision of the prediction. Rapid stability prediction exploiting data from a 4-week-long stability study including temperatures above 40 °C is shown in Fig. 7. In order to demonstrate rapid stability prediction we tried to model stability profiles only based on two weeks data. In case reliable model could not be achieved (*R*^2^ < 0.9, prediction intervals were not verified by measurements or 36 months prediction intervals are wider as generic specifications described above (Table 2)) then additional time points were included until model reliability was reached. That leads to different number of data points per quality attribute shown on Fig. 7 (top panel). The minimal stability study time interval needed to achieve required prediction interval widths^48^ were thus two weeks for sum of acidic variants, three weeks for amount of main variant and four weeks for fragmentation and aggregation (Fig. 7). We speculate that by optimising the study design further we could achieve the same precision of predictions even faster for some quality attributes. For example, first data point for fragmentation at 40 and 25 °C is at 1 month. If data for first and second week time points would be available for these two temperatures, we could probably calculate the prediction interval for mAb1 molecule already after two weeks instead of four weeks as shown on Fig. 7c. Since model predicted increase in fragmentation at two week time point is 0.4% and 1.2% for 25 and 40 °C, respectively, which is above method variability of 0.2%, data from at least 3 temperatures with measurable degradation would be available. This is, as already discussed, enough for a reliable prediction.

## Discussion

Kinetic modelling for predicting long-term stability from accelerated stability data was evaluated for several monoclonal antibodies belonging to IgG1, IgG2 families/types and a fusion protein. Presented results suggest that by applying a degradation model-based on first order kinetics and the Arrhenius equation on accelerated stability data, we can predict long-term (up to 3 year) changes of most relevant stability indicating quality attributes in solution. The highly successful in prediction (above 90% accuracy for 4 different protein molecules) quality attributes include fragments, chemical modifications (acidic variants and main peak), purity, aggregates and potency. Low accuracy is obtained for prediction of basic variants. The reason lies in use of simple first order kinetic model, which cannot cover possible non-monotonic variability of basic variants in time (Figure S4). A cause for more complex kinetic mechanism of basic variants change over time arises possibly from the temperature/pH dependence of succinimide formation^49^.First order kinetic model would fail to describe any non-monotonic changes of any quality attribute. Low prediction accuracy is also obtained for subvisible particles (data not shown), mainly due to high variability of available analytical methods and high complexity of particle formation mechanisms^50–53^ which do not follow Arrhenius kinetics. From a biopharmaceutical application perspective, none of the both quality attributes show any trend at intended storage temperature for investigated molecules and are therefore not shelf-life limiting. For other quality attributes the predictions were successfully determined by using up to 6 month stability data containing three or more temperature conditions (usually 5 °C, 25 °C and 40 °C). The verification of long-term stability predictions at the intended storage temperature of 5 °C was evaluated by comparison of the 95% probability prediction interval and experimental data obtained during the long-term stability studies lasting up to 36 months. Results are summarised in Table 1 for 4 different molecules (mAb1, rituximab, adalimumab and etanercept). Percentage of experimental stability data between 6 and 36 months at 5 °C that are within the 95% probability prediction interval is above 90% for all model fits with goodness of fit *R*^2^ ≥ 0.9. Robustness of the model stability prediction is additionally supported by applying the kinetic modelling to combinations of stability data subsets, which included different temperatures, and analysing the goodness of fit and model parameters. If values for model parameters do not vary between different stability data subsets, this is a strong indication for the appropriateness of the kinetic model and the overall accuracy and robustness of the prediction approach (Fig. 4). Furthermore, by fitting the model with data subsets we can estimate the maximal temperature up to which molecule degradation follows Arrhenius behaviour. The most prominent advantage of using the identified highest temperature storage condition is faster molecule degradation, which allows to conduct a shorter accelerated stability study, which based on our results does not compromise accuracy or robustness of model prediction (Fig. 7). However, to evaluate the associated risk and benefits of very short (weeks) accelerated stability studies, additional research still needs to be done.

Aggregation is a common stability-limiting factor for biologics. It is also known that aggregation is a complex process that in general embraces several aggregation pathways, which are temperature-dependent^9,50^. For mAbs Wang and Roberts^3^ confirmed Arrhenius behaviour in a temperature range from 5 to 40 °C, at higher temperatures the linearity of Arrhenius plot vanished. These findings are in agreement with our data shown on Fig. 4 (panels f, g and h), where at temperatures above 40 °C we found an increase of fitted value for *E*_a_ for all three molecules studied indicating a change in aggregation process. The importance of limiting the temperature range for kinetic modelling of aggregation is demonstrated in Figure S5. Obtained aggregation rates based on up to 40 °C stability data are in a range of 0.1 to 0.2% increase per year and are in agreement with experimental observations. While adding 45 °C data to the modelling dataset results in 100 times slower predicted aggregation rate (Figure S5c). According to the literature, there are notable evidences that aggregation pathways are connected with the conformational stability^54,55^. Our aggregation results (Fig. 4, panels f and g) indeed briefly indicate that the aggregation pathway changes with increasing temperature, which might be linked to the mentioned temperature-induced change in conformational stability. Nevertheless, the presented approach for identification of the maximal temperature up to which aggregation follows the same mechanism as at storage temperature is robust enough to obtain accurate predictions based on simple first order aggregation kinetic model. By selecting the optimal temperature interval we obtained reliable predictions that are verified with experimental data for several mAbs and one fusion protein (Figs. 3a and S1). These two predictions are based on first order degradation kinetic model build on accelerated stability study data with 5 °C to 40 °C conditions. We are aware, however, that even by limiting the temperature range the first order degradation kinetic model does not fully describe the aggregation process during the long-term storage at 5 °C. For example, measured stability data of etanercept at 5 °C (Figure S2b) suggest that the aggregation process involves at least two distinct processes (biphasic change). During the first few months level of aggregates increases with the rate of about 0.4% per year, which drops to about 0.1% per year after 12 months. However, to appropriately model biphasic changes at 5 °C it would be essential to experimentally first detect biphasic behaviour at accelerated conditions and then one can apply two steps model^20^. Therefore some pre-tests are recommended to optimally design temperature conditions and pull points frequency of accelerated stability to experimentally detect biphasic behaviour at several temperatures that is needed to adequately model also biphasic protein aggregation^3^. Nevertheless, for the etanercept molecule shown on Figure S2b, even prediction interval based on first order degradation kinetic model and 6 month accelerated stability study satisfactory describes aggregation, which is verified by long-term stability data.

In the guideline of European Medicines Agency it is stated that the shelf-life and storage conditions of the biologics manufactured for clinical study purposes can be extrapolated for the same period as the experimental data are available but not for more than 12 months (e.g. from six month stability data to a maximum of 12 months), provided that stability studies are conducted in parallel to the clinical studies and throughout its entire duration^13^. Current industrial practice of shelf-life determination is to use linear regression^41^. But in case that the linear regression is complemented in the future by Arrhenius kinetic modelling, similar as for small molecules^12^, applicants would claim 12 months shelf-life already from three months stability data. However it is important to note that selection of kinetic model and the validity of predictions based on Arrhenius kinetic modelling should be first verified for a specific molecule and quality attributes of interest on case by case basis. Predictions could be verified by preliminary studies on non-GMP material using the final primary packaging. The main feature supporting advanced claiming of shelf-life is superior accuracy of Arrhenius modelling in comparison to linear extrapolation. On Fig. 5 (upper panel) we showed an example for one batch of the rituximab molecule. For that batch, based on 3 months accelerated stability study, we predicted sum of aggregates to be below 1% after 12 months of storage at 5 °C and after 36 months below 2%. By using linear extrapolation, predicted intervals for aggregates are much wider, namely below 2.1% at 12 months and below 7.2% at 36 months. Similarly, kinetic Arrhenius modelling leads to more accurate prediction also in case of the sum of acidic variants (Fig. 5, lower panel). More examples of 12 months predictions for the sum of aggregates for different proteins are shown in Figure S3. To generalise, kinetic modelling results in much narrower prediction intervals compared to linear extrapolation, enabling faster, more reliable and robust prediction of stability and shelf-life estimation, both of prime importance in the development of biologics.

Overall, the data presented demonstrate that already accelerated stability studies (up to 6 months) combined with basic first order degradation kinetic model allow for robust and accurate predictions of the long-term (up to 3 years) stability behaviour of monoclonal antibodies at intended storage conditions. The study presented clearly emphasizes that shelf-life model-based prediction can be justified for biologics like monoclonal antibodies, if the stability profile of a product is extensively studied and clearly defined, if state-of-the-art analytical methods are available for the predicted quality attributes, and, of course, if the actual biological molecule in the development is relatively stable. Presented results evidently challenge the current paradigm that stability predictions of biologics from accelerated stability data are not sufficiently robust since molecules are more complex, have distinguishing characteristics and that their molecular integrity, conformation and, hence, biological activity are more sensitive to environmental factors^2,56,57^. Therefore such predictions are not accepted by health authorities for clinical or for marketing authorisation applications^14^ as they are under certain conditions for small molecule drugs ^41,58^. However, the presented results could contribute to regulatory guidelines covering extrapolation principles being updated in the future to acknowledge the use of Arrhenius kinetic modelling for setting the shelf-life of biologics as well. Such as, for example, within existing Breakthrough Therapy (BTD) schemes where the US Food And Drug Association (FDA) already supports the accelerated access to medications indicated for serious or life-threatening conditions with limited treatment options^59^. In addition, recently the European Medicines Agency (EMA) published a draft guideline for PRIME marketing authorisation applications, enhancing EMA support to the development of medicines that target an unmet medical need with the aim to help patients to benefit from these therapies as early as possible^60^. According to the draft guidelines, for a biologic PRIME product, trends in stability data, and therefore the claimed shelf-life, could be extrapolated using predictive stability models generated from prior knowledge of the stability of structurally similar molecules. That is of course different from the proposed Arrhenius strategy, which is based on all the stability data of a specific molecule. Namely, during early development by using non-GMP material one should already (1) select the most appropriate type of kinetic models, (2) optimize accelerated stability study design (temperature conditions, sampling plan), and finally (3) one should verify model by long-term measurements at 5 °C. Guided by acquired knowledge about the specific molecule, a GMP accelerated stability study is further designed and executed, the measurements of which are then used to set up Arrhenius kinetic models for claiming shelf-life. In short, there are however differences in PRIME and Arrhenius approaches but the both proposed strategies pursue the same goal towards approval of shelf-life which is longer than the available product-specific real-time stability data. Taken all together, we believe that the presented findings can contribute towards development and refinement of the regulatory landscape, to allow extrapolation for biologics during the development phase, the clinical phase, and also for marketing authorisation applications in the same way as for small molecules.

## Supplementary Information

Supplementary Figures.
