# Supplementary material for: Long-term stability predictions of therapeutic monoclonal antibodies in solution using Arrhenius-based kinetics
Source: Sci Rep. 2021 Oct 15;11:20534. doi: 10.1038/s41598-021-99875-9 (PMC8519954; doi:10.1038/s41598-021-99875-9)
Supplement: Supplementary file 1 — Supplementary Figures. [file 41598_2021_99875_MOESM1_ESM.docx]

**Supporting information**

**Long-term Stability Predictions of Therapeutic Monoclonal Antibodies in Solution using Arrhenius Based Kinetics**

Drago Kuzman,^1^ Marko Bunc,^1^ Miha Ravnik,^2,3^ Fritz Reiter,^4^ Lan Žagar,^5^ Matjaž Bončina^1,^*

^1^Biologics Drug Product, Technical research and development, Global drug development, Lek d.d., Mengeš, Slovenia

^2^Faculty of Mathematics and Physics, University of Ljubljana, Ljubljana, Slovenia

^3^Josef Stefan Institute, Ljubljana, Slovenia

^4^Regulatory Affairs CMC, Global drug development, Novartis, Kundl, Austria

^5^Revelo d.o.o., Ljubljana, Slovenia

 
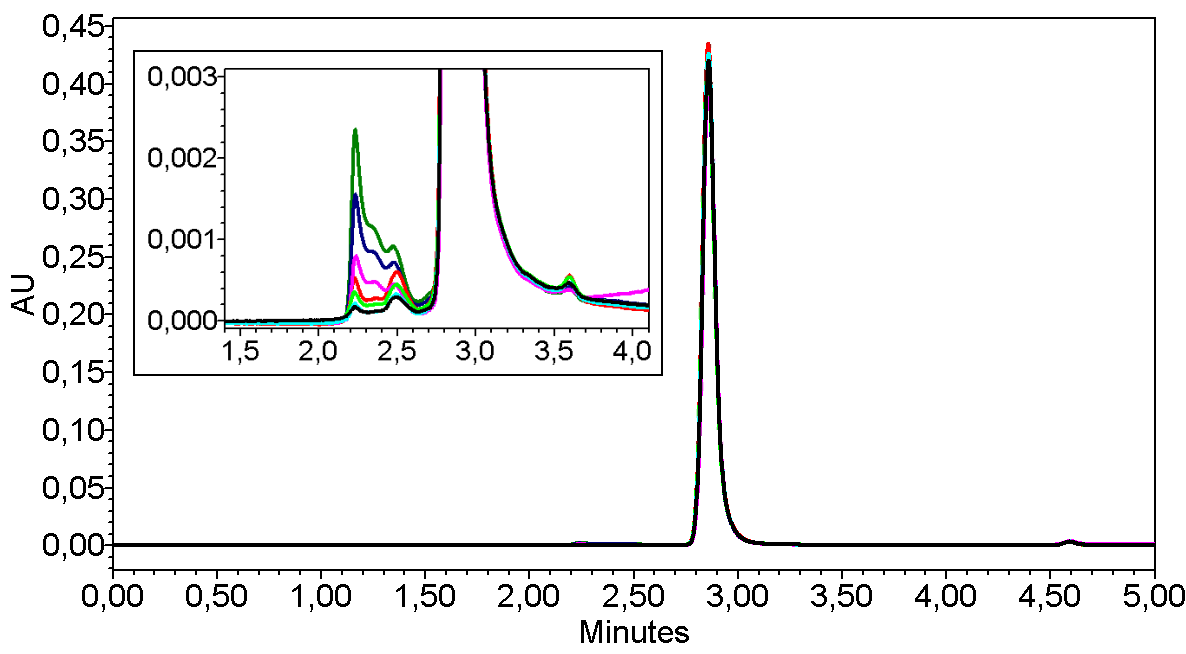


**Figure S1**. Chromatogram overlay of mAb1 samples stored at 25 – 55 °C for different time intervals (up to 1 month) shows area under the curve did not change even at the highest temperature. Black chromatogram shows t0 sample, samples exposed to higher temperatures are in color: light blue – 6 months at 25 °C, light green – 1 month at 35 °C, pink – 3 months at 40 °C, red – 1 month at 45 °C, dark blue – 3 weeks at 50 °C and dark green – 1 week at 55 °C. AU are absorbance units.


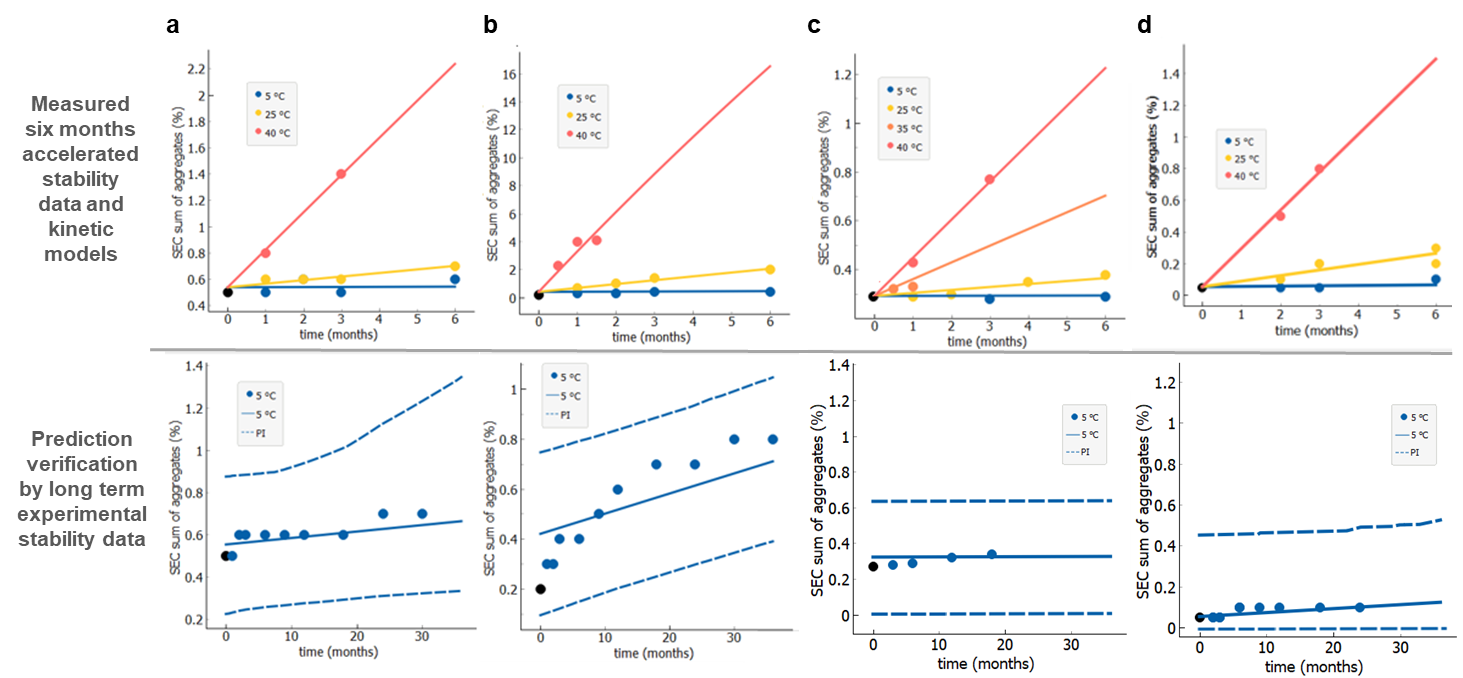


**Figure S2**. Long-term (36 months) prediction of sum of aggregates for adalimumab (a), etanercept (b), mAb1 (c) and denosumab (d). Predictions is based on accelerated stability data (6 months) and kinetic modeling which included Arrhenius temperature dependence of kinetic rates. Accelerated experimental stability data (upper panels, data points) are used to develop the kinetic model to predict long-term stability at intendent storage conditions (lower panels). Mostly, accelerated conditions are 3 months at 40 °C and 6 months are 25 °C. In case of etanercept (b) the duration of 40 °C condition was 1.5 months only. In case of mAb1 (c) the data from 35 °C condition for one month were included. The 95 % prediction interval designated by dashed blues lines is verified by long-term experimental data (lower panels, data points). Measured value at *t* = 0 is designated by black solid circle.


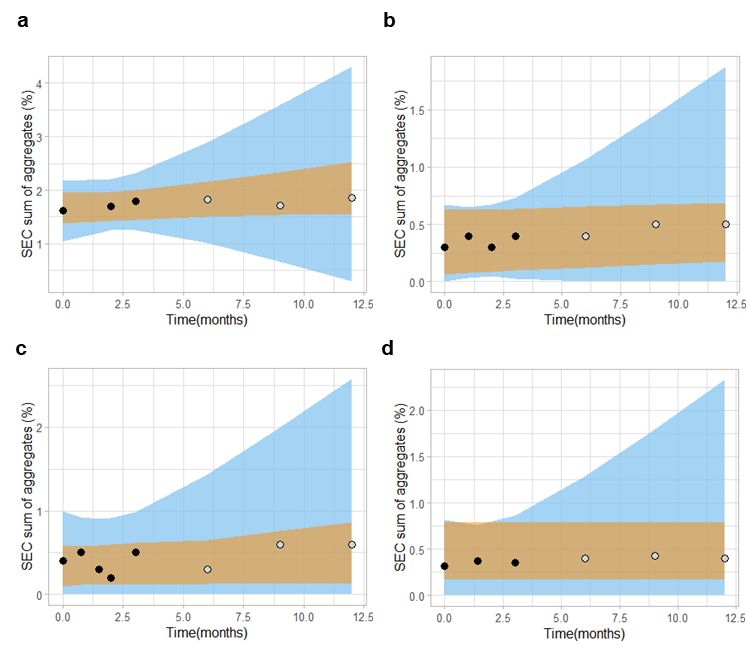


**Figure S3**. Linear (blue) vs Arrhenius kinetic (orange) extrapolation from 3 months stability data (black solid circles) to 12 months of sum of aggregates for selected batches of mAb2 (a), etanercept (b), rituximab (c) and adalimumab (d). Prediction intervals are verified by experimental data from 5 °C storage condition (empty circles).


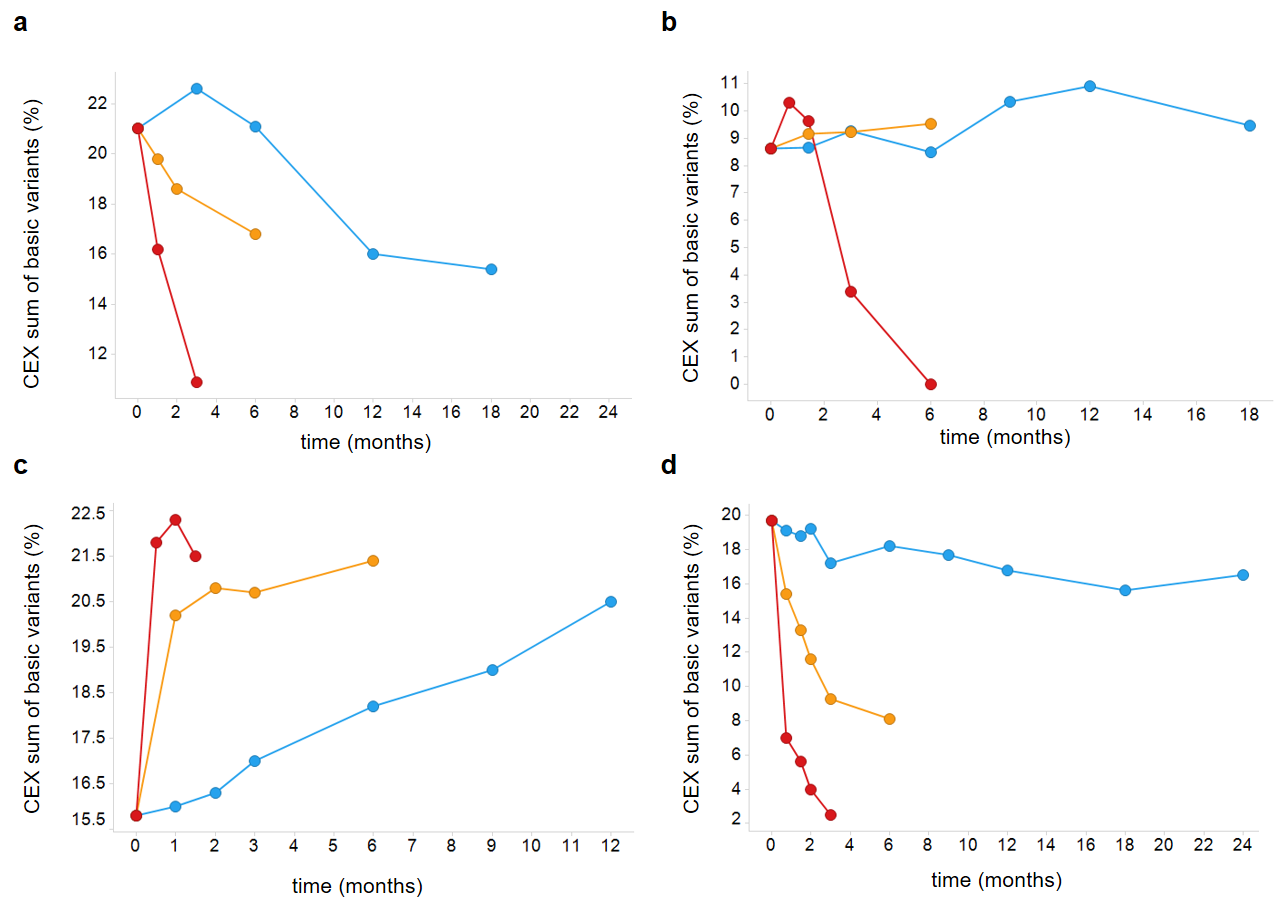


**Figure S4**. Profiles of sum of basic variants profiles as measured at 5°C (blue), 25 °C (orange) and 40 °C (red) for four different molecules (a – mAb1, b – aflibercept, c – etanercept, d – rituximab). Pull points vary due to molecule specific stability protocols. Note non-monotonic behavior on a, b and c panels where initial increase is followed by the decay of the basic variant. The process is faster at higher temperatures. Shown data were measured for one batch with no replicates (N = 1, analytical accuracy SD = 1.0%).


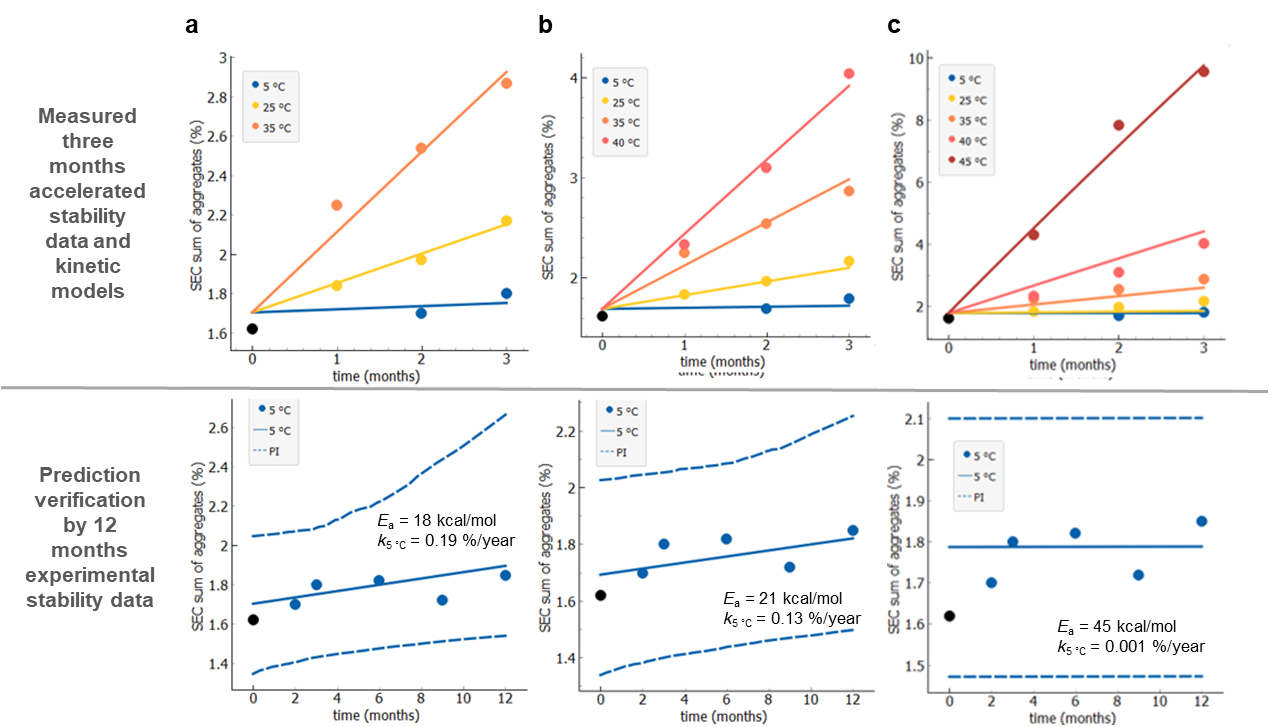


**Figure S5**. Impact of storage temperature on kinetic modeling. Case study for the 12 months prediction of sum of aggregates for mAb2 based on the 3 months stability experiments at accelerated storage conditions as designated by colors (upper panels). The highest temperature condition used for kinetic modeling which included Arrhenius temperature dependence of kinetic rates are 35 °C (a), 40 °C (b) and 45 °C (c). The 95 % prediction interval designated by dashed blue lines is verified by 12 months experimental data (lower panels, data points). For each model fitted activation energy *E*_a_ and calculated degradation rate *k*(*T*_ref_) at 5 °C are shown. Measured value at *t* = 0 is designated by black solid circle.
